# Examining Caregiver- and Family-Level Psychosocial Influences on Child Oral Health Behavioral Outcomes in Racially and Economically Minoritized Urban Families

**DOI:** 10.3390/children11070882

**Published:** 2024-07-21

**Authors:** Sally M. Weinstein, Helen H. Lee, John J. Dziak, Michael L. Berbaum, Tong Zhang, David Avenetti, Anna Sandoval, Molly A. Martin

**Affiliations:** 1 Department of Psychiatry, College of Medicine, University of Illinois Chicago, 1747 W. Roosevelt Road, Chicago, IL 60608, USA; sweins3@uic.edu; 2 Department of Anesthesiology, College of Medicine, University of Illinois Chicago, 1740 W. Taylor Street, Chicago, IL 60612, USA; 3 Institute for Health Research and Policy, University of Illinois Chicago, 1747 W. Roosevelt Road, Chicago, IL 60608, USA; dziak@uic.edu (J.J.D.); mberbaum@uic.edu (M.L.B.); tzhang87@uic.edu (T.Z.); avenetti@uic.edu (D.A.); asando1@uic.edu (A.S.); mollyma@uic.edu (M.A.M.); 4 Department of Pediatric Dentistry, College of Dentistry, University of Illinois Chicago, 801 S. Paulina Street, Chicago, IL 60612, USA; 5 Department of Pediatrics, College of Medicine, University of Illinois Chicago, 840 S. Wood Street, Chicago, IL 60612, USA

**Keywords:** household chaos, oral health promotion, social determinants of health, toothbrushing, psychosocial factors, health disparities, pediatric dentistry

## Abstract

Objectives: Understanding the pathways linking caregiver- and family-level psychosocial factors and child oral health behaviors is critical for addressing oral health disparities. The current study examined the associations between caregiver psychosocial functioning and family chaos and child toothbrushing behaviors in children at high risk for poor oral health outcomes. Methods: Data were drawn from the baseline wave of the CO-OP Chicago Cohort Study (U01DE030067), a longitudinal study on child/caregiver dyads exploring oral health behaviors and caries development in young children (*N* = 296 dyads; child mean age = 5.36, SD = 1.03; caregiver mean age = 33.8 years, SD = 6.70; caregiver race = 43% Black; caregiver ethnicity = 55% Latinx). The oral health behavioral outcomes included child toothbrushing frequency, child plaque levels, and caregiver assistance with child toothbrushing. The data included demographics; caregiver depression, anxiety, post-traumatic stress disorder (PTSD) symptoms, social functioning, social support, and resilience; and family-level household chaos. Results: Multiple regression models indicated that greater household chaos was significantly related to lower caregiver assistance with child toothbrushing (*p* = 0.0075). Additionally, caregiver anxiety and PTSD symptoms as well as number of children in the home significantly predicted higher levels of household chaos (*p* < 0.01). Notably, 18% of caregivers reported clinically significant PTSD. The relationships between caregiver-level psychosocial factors and child oral health behaviors were not significant. Conclusions: The results suggest household chaos may play an important role in child oral health behaviors and highlight the importance of investigating family-level factors for understanding and addressing child oral health risk.

## 1. Introduction

Significant disparities in child oral health exist, with the prevalence and severity of dental caries—and associated morbidity—greatest among low-income and minority children [1,2,3]. Research, guided by social-ecological models of health [4,5], is increasingly moving beyond traditional models of risk to examine multi-level (i.e., individual, family, community, and public health) risk and protective factors that contribute to oral health behaviors. However, insight into how and why these factors operate is still limited. A greater understanding of the pathways between psychosocial factors, social determinants, and pediatric oral health outcomes is necessary for developing targeted interventions and policies to address such disparities. 

Families are key contexts for establishing oral health-promoting behaviors [6]. Pediatric dental guidelines recommend caregiver assistance with child oral health behaviors [7]; however, our past work has shown that stressors influencing caregivers may hinder this assistance [8,9]. Thus, identifying modifiable risk and protective factors within the family context may inform practices to improve child outcomes among those at greatest risk. The research overall supports a relationship between caregiver psychosocial difficulties and poorer child oral health [10]. In a large prospective study, Auger and colleagues found that children of mothers with mental health diagnoses (depression, stress and anxiety disorder, psychotic disorder, and personality disorders) had a greater risk of caries as compared to children of women with no mental health diagnoses, with associations persisting beyond age 7 [11]. Several studies have demonstrated caregiver depression specifically as a risk factor for child caries development [12,13] and risk-related behaviors such as lower child toothbrushing frequency and increased likelihood to lack routine child dental care in the past year [14], even after adjusting for demographic and socioeconomic variables. Similarly, caregiver depression negatively impacted the response to a caregiver-focused pediatric dental intervention [15]. Studies have also shown associations between maternal anxiety symptoms and child caries [16,17]. Few studies have investigated the relationships between caregiver post-traumatic stress disorder (PTSD) and child oral health, but extant findings suggest a possible link. Greater caregiver life stressors differentiated between children with and without caries [18], and prenatal psychological stress among Iraqi mothers during a period of sociopolitical upheaval predicted higher odds of hypomineralized enamel defects in children [19]. In contrast, protective factors such as caregiver social support have been associated with fewer dental caries in children [20,21].

Together, these findings underscore the importance of considering caregiver mental health in child oral health research and practices, and they informed the goals of the current study. Yet, the literature is mixed, not always demonstrating clear relationships [22,23], and it does not include trauma or resilience, suggesting we still have much to learn about how psychosocial factors contribute to oral health disparities. These disparities are also impacted by factors operating on multiple levels [24], but the relative influences of caregiver- and family-level psychosocial factors on child oral health behavioral outcomes among high-risk groups have not been described. To build on previous research establishing the importance of psychosocial factors for child oral health [10,11,12,13,14,15,16,17,18,19] and also address existing gaps in the literature, the current study examined if and how caregiver- and family-level psychosocial factors, including indices of caregiver mental health, family structure, and household chaos, are associated with worse child oral health behaviors. Our prior work examining urban and racially and economically minoritized children 6–36 months old [8,9] indicated that youth with frequent caregiver help with toothbrushing had statistically significantly lower plaque levels than youth without help. We expand on past work in the present study by exploring the caregiver and family factors that may promote or interfere with caregiver assistance with brushing and other oral health behaviors.

Guided by the literature [10,11,12,13,14,15,16,17,18,19], we hypothesized that psychosocial factors at the caregiver level (i.e., higher depression, anxiety, and PTSD symptoms, as well as lower social support/functioning and lower resilience) and family level (higher household chaos) would be associated with worse toothbrushing behaviors. We examined these questions among a study group of racially and economically minoritized families in the Chicagoland area at high risk for poor oral health outcomes as well as psychosocial difficulties [25,26]. Given that the prevalence of child caries in Chicago exceeds national rates [27], the Chicago area presents a particularly rich setting for examining the oral health disease burden in children. Such knowledge is essential for developing effective interventions to remedy existing health disparities. 

## 2. Materials and Methods

### 2.1. Study Design and Participants

The Coordinated Oral Health Promotion (CO-OP) Chicago Cohort Study (U01DE030067) is a longitudinal cohort study building upon the CO-OP Chicago cluster-randomized trial (NCT03397589) (see [8,9]) to explore how multi-level factors influence oral health behaviors and caries development in children over time using a community-based participatory research (CBRP) approach [28]. The CO-OP Chicago Cohort Study participants include child/caregiver dyads from the CO-OP Chicago trial as well as child/caregiver dyads recruited at partner community clinics and Special Supplemental Nutrition Program for Women, Infants, and Children (WIC) centers. Families are excluded if the child is in foster care or is a ward of the state or if the family does not speak English or Spanish. Data collection occurs every six months, mainly in participant homes, and families are followed for up to four years. Data for the current study were drawn from the completed baseline wave. Research assistants (RAs) verbally ask study questions using visual prompts to show response options and record answers electronically. Each visit lasts approximately one hour. The caregivers receive $40 after completion of the baseline data collection visit. Data for the current study included only the baseline cohort (*N* = 332 caregiver/child dyads). 

### 2.2. Measures

#### 2.2.1. Demographics and Household Characteristics

We collected child and caregiver demographic information related to age (years), reported sex (male or female), race and ethnicity, residential address, family structure (total number of children under 18 years old in the household), caregiver relationship status, household income, and caregiver education. 

#### 2.2.2. Caregiver-Level Psychosocial Factors

Caregiver psychosocial factors were assessed using Patient-Reported Outcomes Measurement Information System (PROMIS) measures [29]. The specific domains included depression, anxiety, social support (emotional, informational, and instrumental), and social functioning (ability to participate in social roles and activities). Resilience was assessed using the Brief Resilience Scale [30]. Post-traumatic stress disorder (PTSD) symptoms were assessed via the six-item Short Form of the PTSD Checklist—Civilian Version [31], and this measure is well-validated for its use in trauma research and screening in primary care settings, with suggested clinical cutoffs (>13). Following Lang and Stein, 2005 [31], scores of 14 and above were categorized as clinically significant levels of PTSD symptoms. 

#### 2.2.3. Family-Level Psychosocial Factors

The Confusion, Hubbub, and Order Scale (CHAOS) is a validated measure of household chaos, including the level of commotion, disorganization, and routine in the home (e.g., “We are usually able to stay on top of things”) on a yes/no scale [32]. Note that one item was modified for our study (“It’s a real zoo in our home” reworded as “It is authentically chaotic in our home”). Higher scores reflect greater chaos and disorganization. The CHAOS scale has strong validity, reliability, and stability over a twelve-month period and characterizes chronic family conditions [32]. 

#### 2.2.4. Oral Health Behaviors

RAs asked caregivers about (1) the frequency of their child’s toothbrushing and (2) caregiver assistance with brushing [33]. As a measure of brushing quality, child plaque levels were obtained. To capture plaque, a plaque disclosing solution was painted on children’s primary maxillary incisors by the RAs, and teeth were photographed before and after the application. Disclosed images were asynchronously scored by calibrated clinicians using the Oral Hygiene Index-Maxillary Incisor Simplified (OHI-MIS) scale, which was modified from the simplified Oral Health Index [34], and plaque was scored from 0–3 based on the extent of surface areas covered. A score of 0 = no plaque, 1 = plaque on 1/3 of the incisor, 2 = plaque on >1/3 but <2/3 of the incisor, and 3 = plaque on >2/3 of the tooth surface. The plaque score was calculated as an average over non-missing teeth and treated as a continuous variable. Clinician calibration was performed annually and evaluated interrater reliability to a gold standard examiner (must meet a threshold of 0.75 for the Lin’s Concordance) as well as intra-rater reliability over time [35]. 

### 2.3. Analytic Approach

To explore interrelationships among caregiver- and family-level psychosocial constructs and oral health behavioral outcomes and to identify possible overlaps in constructs, we first calculated Spearman correlation coefficients (to allow for non-normally distributed variables). We then fit a series of regression models examining caregiver- and family- level psychosocial factors as predictors of (1) child toothbrushing frequency, (2) child plaque score, and (3) caregiver assistance with child toothbrushing. Plaque score was treated as a continuous variable. Child toothbrushing frequency was dichotomized as twice a day or more versus less than twice a day because twice daily brushing is the standard recommendation. Caregiver assistance with toothbrushing was dichotomized as yes (most of the time/always) or no (never/some of the time). All caregiver- and family-level psychosocial variables were included together in models to examine the relative contributions of each predictor on oral health behaviors, controlling for all other factors. As a secondary analysis to better characterize the relationships among caregiver- and family-level factors, we examined caregiver psychosocial factors as predictors of the family-level factor household chaos. The analyses used multiple linear regression models for continuous outcomes (household chaos and plaque scores) and multiple logistic regression for binary outcomes (child toothbrushing frequency and caregiver assistance with child toothbrushing). All models controlled for child age, caregiver race (Black, yes/no), and caregiver ethnicity (Latinx, yes/no) given that these variables have been previously associated with oral health behaviors, and caregiver gender was not included as nearly all caregivers identified as female. Complete case analyses were implemented as the proportion of missing data was negligible. Residuals from all models were evaluated for normality and outliers, and no departures from normality or outliers were identified.

## 3. Results

### 3.1. Participant Demographics and Oral Health Behaviors

The caregivers were predominantly female and identified as primarily Black in race or Latinx in ethnicity (Table 1). Approximately half the caregivers had achieved a high school diploma/GED or less (50.3%). A majority of the participants (75.8%) reported household incomes of below $50,000. Notably, high rates of suboptimal child toothbrushing frequency (i.e., less than the recommended twice/day) were reported (33.1%), and less than half of children (41.8%) had consistent caregiver help with toothbrushing. The mean plaque score was 1.56 (SD = 0.39), and scores of 0.7–1.8 reflected fair and 1.9 or higher reflected poor oral hygiene [36]. 

### 3.2. Psychosocial Characteristics

The psychosocial characteristics at the caregiver and family level are provided in Table 2. The caregivers reported elevated levels of PTSD symptoms (18% met the clinical screening criteria for PTSD) and below average levels of social functioning, although the levels of all other psychosocial factors were within the normal range. The mean household chaos scores were low but comparable to other studies [37,38] and the original validation study [32]. 

### 3.3. Correlation Analyses

Spearman correlation coefficients examined the relationships among the psychosocial variables and oral health behaviors (toothbrushing frequency, caregiver assistance with toothbrushing, and plaque score) and are shown in Table 3. Interrelationships among the caregiver-level psychosocial variables suggested related but largely distinct constructs, with the largest correlations found for caregiver depression and anxiety symptoms (*r* = 0.72) and emotional and informational support (*r* = 0.76). Similarly, household chaos was related to but distinct from all caregiver psychosocial factors, with the exception of caregiver resilience. The correlations between the caregiver-level psychosocial variables and oral health behaviors (toothbrushing frequency, plaque levels, and caregiver assistance with toothbrushing) were nonsignificant. Household chaos was significantly and inversely related to caregiver assistance with toothbrushing.

### 3.4. Regression Models

To test our primary aims, we fit a series of regression models to explore the caregiver- and family-level psychosocial predictors of oral health behavioral outcomes, including (1) child toothbrushing frequency, (2) child plaque level, and (3) caregiver assistance with child toothbrushing, as specified above. The results of the multiple logistic regression model examining the first outcome, child toothbrushing frequency, including the estimated coefficients, the odds ratios (ORs), and the 95% confidence intervals (CIs), are shown in Table 4, and the effects for caregiver psychosocial functioning and household chaos were nonsignificant. We note that a proportional odds ordinal logistic model was also separately fit to predict the outcome as a four-level ordinal value, but the results were similar and, thus, only the logistic regression is shown. 

Next, we examined child plaque score as the outcome (Table 5). Emotional and informational support were significant predictors of child plaque, suggesting that lower levels of emotional support but higher levels of informational support were associated with higher (i.e., worse) child plaque scores. However, the high degree of collinearity between these support indices (*r* = 0.76 and *p* < 0.001) suggested that the significant effects were likely spurious, rendering interpretation difficult. No other caregiver- or family-level predictors were significant. 

Finally, a multiple logistic regression model examined caregiver assistance with child toothbrushing (Table 6). The findings indicated a significant effect for household chaos, adjusting for caregiver PTSD and anxiety symptoms, family structure, and demographic controls such that higher household chaos was associated with lower odds of caregiver help with toothbrushing. Child age was also significant, indicating that older children received less assistance with toothbrushing. Again, a proportional odds ordinal logistic model was also separately fit to predict the outcome as a four-level ordinal variable, with similar results obtained. 

To better understand if a family-level factor, namely, household chaos, could be operating as an intermediate outcome, we examined a linear regression model of caregiver-level psychosocial predictors of household chaos. Family composition, including structure (number of children in the home) and caregiver relationship status were also included as predictors of household chaos, guided by the literature [39]. The findings, shown in Table 7, indicated that caregiver anxiety, caregiver PTSD, and family structure (number of children in the home) were significantly related to household chaos, controlling for any effects of other psychosocial factors and demographics. These findings suggest that higher levels of caregiver anxiety and PTSD symptoms, as well as more children in the household, are associated with higher levels of household chaos.

**Figure 1:**
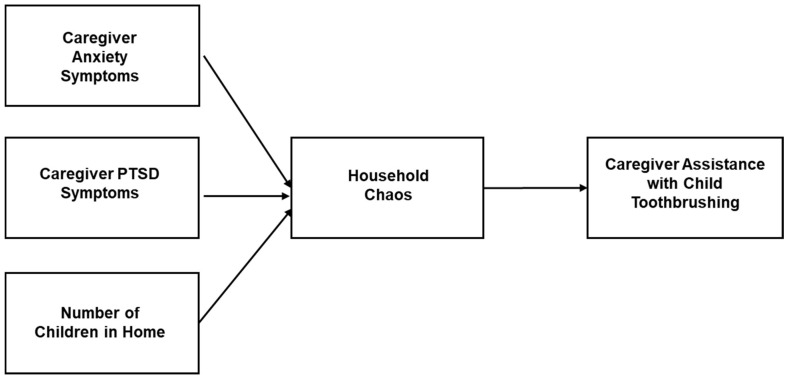
Conceptual model of the significant caregiver- and family-level influences on child oral health behaviors (caregiver assistance with child toothbrushing).

To illustrate our findings, the proposed relationships identified in our models examining caregiver- and family-level factors and child oral health are depicted in Figure 1. Our findings suggest a possible pathway whereby caregiver anxiety and PTSD symptoms predict higher levels of household chaos, and higher chaos, in turn, is associated with lower odds of caregiver assistance with toothbrushing. We note that this model represents only a conceptual illustration of the findings, and it is not a formal mediation or path analyses model.

## 4. Discussion

The current study examined caregiver- and family-level psychosocial influences on child oral health behaviors among a study group that was at high risk for poor oral health outcomes. A third of the children in this study reported less than the recommended toothbrushing frequency, and consistent caregiver help with toothbrushing was low. Moreover, the prevalence of caregiver PTSD greatly exceeded national rates [40]. The results indicated that household chaos was significantly related to caregiver assistance with child toothbrushing. Additionally, caregiver anxiety and PTSD symptoms, as well as the number of children in the home, significantly predicted higher levels of household chaos. The findings partially supported our hypotheses, suggesting that household chaos, a family-level factor, may play an important and potentially modifiable role in oral health disparities. However, our results differed from studies demonstrating caregiver stress, anxiety, and depression as risk factors for child oral health, e.g., [11,12,13,14,15,16,17]. Household chaos may also serve as a proximal pathway through which caregiver psychosocial risk factors (caregiver anxiety and PTSD) as well as unmodifiable factors (family structure) influence child oral health behaviors (as illustrated in Figure 1). These relationships were examined cross-sectionally, and the results did not support an overall link between caregiver mental health factors and child oral health behavioral outcomes. Thus, we did not test mediation, and the interpretations were made cautiously. Nonetheless, the results highlight the importance of further investigations on family-level factors for understanding child oral health risk within a social ecological framework.

Higher levels of household chaos were associated with lower odds of caregiver assistance with child toothbrushing, although they were not associated with child toothbrushing frequency or plaque levels. Caregiver assistance with brushing is recommended by pediatric dental guidelines, given evidence that assistance is key for promoting child oral health and has been demonstrated to relate to fewer caries [41]. Younger children do not possess adequate manual dexterity for effective brushing [42], but this practical need for help often clashes with caregiver desires to promote autonomy in brushing. Along these lines, child age was also a predictor of caregiver assistance, with older children receiving less caregiver assistance with toothbrushing. Our past longitudinal work has shown that improvements in caregiver assistance over time predict improvements in child plaque scores [9]. Additional longitudinal research is needed to understand how caregiver assistance with brushing relates to changes in child plaque scores and the development of caries over time. 

Across the analyses, the findings overall did not support direct relationships between the caregiver psychosocial factors (depression, anxiety, or PTSD symptoms; social support/functioning; and resilience) and child toothbrushing frequency, plaque levels, or caregiver assistance with toothbrushing. We did observe a possible signal between caregiver social support and child plaque levels, with the effects suggesting that lower emotional support but higher informational support was associated with higher plaque levels; however, given the high collinearity between these variables, the interpretation of the findings was difficult and warrants further investigation. Although our findings were inconsistent with prior literature demonstrating that caregiver depression and anxiety relate to child oral health risk, the existing literature is quite mixed [10], and no work has directly examined caregiver PTSD symptoms in relation to child oral health. Our findings were consistent with studies that reported nonsignificant associations between caregiver psychosocial factors and child oral health outcomes [22,23]. Heterogeneity in the oral health behaviors and outcomes examined, in the assessment of caregiver mental health, and in study group composition across studies may contribute to inconsistencies in findings. A majority of the caregivers in this study did not have elevated levels of depression and anxiety symptoms, which possibly limited our ability to capture their impacts on child oral health. Our instruments also may have not adequately described the psychosocial experiences of racially and economically marginalized populations, where symptoms of depression and anxiety may operate on different scales or require modified terminology to be captured [43]. Beyond such measurement variability, however, the relationships between individual-, caregiver-, and family-level factors and child oral health behaviors and outcomes are highly complex and transactional [15], and future longitudinal studies are warranted to characterize the nature of these associations. Indeed, our study suggests that caregiver mental health may be less relevant than family-level factors for child oral health behaviors. Moreover, caregiver-level factors may not have direct effects on child oral health but rather exert influence via more proximal pathways such as household chaos, as caregiver anxiety and PTSD symptoms significantly predicted chaos levels.

Household chaos is increasingly recognized as a key construct to consider in health research and policy [44], and the current findings were consistent with this burgeoning literature. Distinct from other measures of family functioning, caregiver stress, or family socioeconomic status, household chaos uniquely captures the high levels of stimuli or noise, unpredictability, and lack of structure in the home environment [32,39]. To our knowledge, this is the first study to examine the relationship between chaos and child oral health behaviors. However, much research has established relationships between household chaos and adverse outcomes across domains, age, disease status, and socioeconomic status factors, including child and parent psychosocial functioning, child cognitive and academic outcomes, and child health behaviors [39]. Several studies have shown that chaos negatively impacts child health outcomes and the management of chronic health conditions. In past work examining youth with uncontrolled asthma, we found that chaos corresponded to worse asthma control as well as less parental help with asthma medication [38]. Additionally, chaos is associated with poorer glycemic control in youth with type 1 diabetes mellitus [45], infant obesity risk [46], and worse general child health [47].

Our current study suggests that greater chaos in the home may interfere with caregiver help with child brushing. Higher levels of chaos are associated with adverse effects on parenting behaviors and parent–child interactions across studies, and adverse parenting behaviors have also been highlighted as a mediator of household chaos effects on child outcomes [39]. As such, caregiver management and control of consistent daily routines may be compromised in higher-chaos households, including routines around toothbrushing as well as caregiver availability during child brushing routines. These results were consistent with our prior findings where caregiver assistance with child brushing may have been impeded by stressors on caregiver time or chaotic brushing routines [9]. 

The findings also indicated that higher levels of caregiver anxiety and PTSD symptoms, as well as more children in the household, were associated with greater levels of household chaos, controlling for all other demographic and psychosocial factors. Causal relationships could not be established in our cross-sectional study, but past research indicates that individuals with significant anxiety and trauma symptoms experience higher levels of arousal and dysregulated mood and may also have difficulty with executive functioning [48]. This constellation of symptoms could place significant strain on a caregiver’s ability to effectively maintain predictability, structure, and routines—and minimize disorganization or commotion—in the home. Although chaos was not established as a mediator in these cross-sectional analyses and caregiver psychosocial functioning did not directly relate to oral health behaviors in our work, our results offer promise for future longitudinal studies in exploring chaos as a possible mediating pathway. Chaos has been shown to mediate the relationships between caregiver mental health and various child outcomes, including child asthma control [38] and negative child psychosocial outcomes [49], as well as to moderate risk–outcome relationships such that risk factors have the greatest impacts on child outcomes in more chaotic homes [39].

The strengths of the study include its focus on high-risk, racially and economically marginalized families; the use of data from both children and caregivers; and the use of standardized instruments that capture a range of domains in mental health and both self-reported and observed oral health behaviors. The limitations must also be noted. We explored a conceptual model for informing future research, but our examination of the direction of the effects and causality were precluded by the study’s cross-sectional design. Our measures of mental health did not provide clinical diagnoses, and our findings may not be generalized to clinical populations; additionally, our caregiver psychosocial functioning measures may not have adequately captured caregiver symptomatology in our study group (as noted in [43]). In addition, the current study examined child toothbrushing behaviors but did not examine predictors of oral disease. Future work must examine how psychosocial factors, including household chaos, confer risk for childhood caries. Further, our focus on racially and economically minoritized families limits the generalizability of findings to other populations. The context of race/ethnicity and culture needs to be considered in future work, as well as more systemic factors such as structural racism. It will be important for future study designs to allow for comparisons of groups that represent the ethnic and racial diversity of the greater United States population. 

## 5. Conclusions

Our findings have important implications for future research and practices aimed at reducing oral health disparities. Longitudinal investigations of household chaos and connections between caregiver mental health and child oral health risk are warranted. Future intervention research is also needed to address household-level factors and their impacts on child oral health behaviors and plaque levels. Social determinants of oral health, such as poverty, access to care, and immigrations status, are consistently linked to health disparities [2,25], but they can be difficult to modify in impactful ways. Identifying family-level behaviors that can be modifiable targets for intervention is therefore a public health priority. The results suggest that increased attention to developing structured, consistent family toothbrushing routines and problem-solving difficulties with caregiver help with child brushing may be particularly relevant among families with higher levels of chaos, especially in the context of caregiver anxiety and PTSD symptomatology, as well as larger families, to minimize oral health risk. Screening for household chaos and caregiver trauma and anxiety symptoms via the brief self-report measures used in this study (e.g., [31,32]) or other readily available measures (e.g., GAD-7 [50]) can be flexibly integrated in routine practice settings. Such efforts would illuminate areas of household risk and guide clinical discussion and decision-making to promote optimal oral health behavior.

## Figures and Tables

**Table 1:** Participant demographic and oral health characteristics.

**Child Characteristics**	
Child female gender, *n (%)* (*N =* 332)	158 (47.6%)
Age (years), mean (SD) (*N =* 332)	5.36 (1.03)
Child medical insurance source, *n (%)* (*N =* 327)	
Medicaid	302 (91.8%)
Other	25 (7.6%)
No health insurance	2 (0.6%)
Child dental insurance source, *n (%)* (*N =* 328)	
Medicaid	283 (86.3%)
Other	29 (8.8%)
None	16 (4.9%)
Toothbrushing frequency, *n (%)* (*N =* 329)	
Less than twice/day	109 (33.1%)
Twice/day or more	220 (66.9%)
Caregiver assistance with brushing, *n (%) (N = 330)*	
Never or sometimes	192 (58.2%)
Most of the time or always	138 (41.8%)
Plaque score, mean (SD)	1.56 (0.39)
**Caregiver Characteristics**	
Age (years), mean (SD)	33.8 (6.7)
Female gender, *n (%)* (*N =* 332)	320 (96.4%)
Ethnicity (Latinx), *n (%)* (*N =* 330)	181 (54.9%)
Race (Black), *n (%)* (*N =* 332)	144 (43.4%)
Relationship to child, *n (%)* (*N =* 332)	
Parent	327 (98.5%)
Grandparent	1 (0.3%)
Other relative	4 (1.2%)
Caregiver highest degree earned, *n (%)* (*N =* 330)	41 (12.4%)
Less than high school	125 (37.9%)
High school/GED	164 (49.7%)
More than high school	
Household income in last year, *n (%)* (*N =* 281)	
<$20,000	93 (33.1%)
$20,000–49,999	120 (42.7%)
$50,000–69,999	36 (12.8%)
$70,000 or more	32 (11.4%)
Caregiver relationship status, *n (%)* (*N* = 329)	
Single	142 (43.2%)
Married/partner	165 (50.1%)
Living separately from spouse/partner	4 (1.2%)
Widowed/divorced	18 (5.5%)
Total children in household, mean (SD)	2.57 (1.36)

**Table 2:** Caregiver/family psychosocial characteristics.

Caregiver/Family Psychosocial Characteristics	
Post-traumatic stress disorder (PTSD) in clinical range (score > 13), *n* (%) (*N =* 316)	57 (18%)
Depression symptoms (T-score), mean (SD) (*N =* 328)	46.66 (7.84)
Anxiety symptoms (T-score), mean (SD) (*N =* 328)	47.14 (8.71)
Emotional support (T-score), mean (SD) (*N =* 326)	55.78 (9.13)
Instrumental support (T-score), mean (SD) (*N =* 325)	57.99 (10.97)
Informational support (T-score), mean (SD) (*N =* 324)	54.26 (10.42)
Social functioning (T-score), mean (SD) (*N =* 330)	33.69 (7.25)
Brief resilience scale, mean (SD) (*N =* 327)	3.00 (0.42)
Household chaos, mean (SD) (*N =* 326)	2.22 (0.64)

**Table 3:** Spearman correlations among the caregiver- and family-level psychosocial variables and child oral health behaviors.

	1	2	3	4	5	6	7	8	9	10	11	12
1. Depression	**---**											
2. Anxiety	**0.72**	**---**										
3. Support - emotional	**−0.36**	**−0.35**	**---**									
4. Support - information	**−0.35**	**−0.31**	**0.76**	**---**								
5. Support - instrument	**−0.29**	**−0.25**	**0.60**	**0.58**	**---**							
6. Social functioning	**0.46**	**0.52**	**−0.27**	**−0.22**	**−0.28**	**---**						
7. PTSD	**0.42**	**0.45**	**−0.32**	**−0.23**	**−0.22**	**0.36**	**---**					
8. Resilience	**0.12**	**0.15**	0.05	0.08	0.05	0.09	**0.16**	**---**				
9. Chaos	**0.24**	**0.29**	−0.14	**−0.12**	**−0.18**	0.24	**0.28**	0.08	**---**			
10. Child plaque score	0.00	−0.01	−0.02	0.06	0.06	−0.05	−0.02	−0.06	0.05	**---**		
11. Child brushing frequency	−0.02	−0.04	0.04	0.01	0.04	−0.05	−0.04	0.06	**−0.10**	−0.02	**---**	
12. Caregiver assistance with brushing	0.01	0.01	0.00	0.02	0.08	−0.01	−0.06	−0.02	**−0.17**	0.00	0.00	**---**

Abbreviation: PTSD, post-traumatic stress disorder. Note: the numbers 1–12 (top row) correspond to the variables as listed in the first column. The bolded and underlined values reflect *p* < 0.05.

**Table 4:** Multiple logistic regression model predicting the dichotomized brushing frequency (twice or more daily vs. less than twice daily) from the demographic and psychosocial factors, with *N* = 305.

				Odds Ratio	
Parameter	Estimate	Std. Err.	*p*	Estimate	95% CI
Intercept	0.4958	1.9405	0.7983		
Child age	0.0079	0.1243	0.9494	1.008	(0.790, 1.286)
Caregiver race	−0.3939	0.5745	0.4929	0.674	(0.219, 2.079)
Caregiver ethnicity	−0.0143	0.5698	0.9800	0.986	(0.323, 3.012)
Caregiver depression	0.0029	0.0257	0.9097	1.003	(0.954, 1.055)
Caregiver anxiety	−0.0083	0.0235	0.7245	0.992	(0.947, 1.039)
Caregiver PTSD	−0.0085	0.0213	0.6905	0.992	(0.951, 1.034)
Emotional support	0.0221	0.0251	0.3783	1.022	(0.973, 1.074)
Informational support	−0.0219	0.0197	0.2664	0.978	(0.941, 1.017)
Instrumental support	0.0028	0.0153	0.8565	1.003	(0.973, 1.033)
Social functioning	0.0000	0.0213	0.9994	1.000	(0.959, 1.043)
Caregiver resilience	0.4095	0.2990	0.1709	1.506	(0.838, 2.706)
Household chaos	−0.3023	0.2063	0.1427	0.739	(0.493, 1.107)

Abbreviation: PTSD, post-traumatic stress disorder.

**Table 5:** Multiple linear regression model predicting child plaque score from the demographic and psychosocial factors, with *N* = 299.

Parameter	Estimate	Std. Err.	*p*
Intercept	1.8117	0.3652	<0.001
Child age	0.0347	0.0230	0.1324
Caregiver race	−0.1732	0.1070	0.1065
Caregiver ethnicity	−0.1548	0.1060	0.1454
Caregiver depression	−0.0006	0.0047	0.8947
Caregiver anxiety	0.0006	0.0043	0.8863
Caregiver PTSD	−0.0005	0.0040	0.9062
**Emotional support**	**−0.0121**	**0.0046**	**0.0091**
**Informational support**	**0.0072**	**0.0036**	**0.0437**
Instrumental support	0.0040	0.0028	0.1565
Social functioning	−0.0068	0.0040	0.0864
Caregiver resilience	−0.0384	0.0556	0.4906
Household chaos	0.0512	0.0380	0.1789

Note: model R^2^ = 0.06. Abbreviation: PTSD, post-traumatic stress disorder.

**Table 6:** Multiple logistic regression model predicting caregiver assistance with toothbrushing from the demographic and psychosocial factors, with *N* = 307.

				Odds Ratio	
Parameter	Estimate	Std. Err.	*p*	Estimate	95% CI
Intercept	3.1992	1.9641	0.1033		
**Child age**	**−0.5326**	**0.1270**	**<0.0001**	**0.587**	**(0.458, 0.753)**
Caregiver race	0.4009	0.5753	0.4859	1.493	(0.484, 4.611)
Caregiver ethnicity	−0.0732	0.5698	0.8978	0.929	(0.304, 2.839)
Caregiver depression	0.0058	0.0258	0.8234	1.006	(0.956, 1.058)
Caregiver anxiety	0.0168	0.0236	0.4751	1.017	(0.971, 1.065)
Caregiver PTSD	−0.0272	0.0218	0.2120	0.973	(0.933, 1.016)
Emotional support	−0.0125	0.0248	0.6152	0.988	(0.941, 1.037)
Informational support	0.0061	0.0192	0.7522	1.006	(0.969, 1.045)
Instrumental support	0.0132	0.0155	0.3937	1.013	(0.983, 1.044)
Social functioning	0.0086	0.0215	0.6908	1.009	(0.967, 1.052)
Caregiver resilience	−0.3747	0.3027	0.2157	0.687	(0.380, 1.244)
**Household chaos**	**−0.5683**	**0.2126**	**0.0075**	**0.566**	**(0.373, 0.859)**

Abbreviation: PTSD, post-traumatic stress disorder.

**Table 7:** Multiple linear regression model predicting family-level household chaos from the caregiver-level psychosocial factors and demographic controls, with *N* = 304.

Parameter	Estimate	Std. Err.	*p*
Intercept	0.8190	0.5356	0.1273
Child age	0.0403	0.0332	0.2257
Caregiver race	−0.0940	0.1594	0.5560
Caregiver ethnicity	−0.1140	0.1569	0.4683
Caregiver relationship status	−0.0187	0.0737	0.8002
**Total children in household**	**0.1481**	**0.0255**	**<0.001**
Caregiver depression	0.0025	0.0070	0.7189
**Caregiver anxiety**	**0.0141**	**0.0063**	**0.0261**
**Caregiver PTSD score**	**0.0125**	**0.0058**	**0.0316**
Emotional support	0.0048	0.0067	0.4747
Informational support	0.0009	0.0053	0.8615
Instrumental support	−0.0071	0.0041	0.0848
Social functioning	0.0046	0.0058	0.4260
Caregiver resilience	−0.0166	0.0812	0.8385

Note: model R^2^ = 0.22. Abbreviation: PTSD, post-traumatic stress disorder.

## Data Availability

The data presented in this study are available on request from the corresponding author. The data are not publicly available due to the ongoing nature of the longitudinal study, Data will be publicly available at the study’s conclusion.
